# Homozygous Phosphatidylinositol Glycan Class T Mutation in an Indian Girl With Multiple Congenital Anomalies-Hypotonia-Seizures Syndrome 3

**DOI:** 10.7759/cureus.14727

**Published:** 2021-04-28

**Authors:** Dudipala Sai Chandar, Battu Krishna Chaithanya, Mandapuram Prashanthi

**Affiliations:** 1 Pediatric Neurology, Star Women and Children Hospital, Karim Nagar, IND; 2 Pediatrics, Prathima Institute of Medical Sciences, Karim Nagar, IND; 3 Pediatrics, Star Women and Children Hospital, Karim Nagar, IND

**Keywords:** hypotonia, infantile onset seizures, dysmorphic features

## Abstract

Multiple congenital anomalies-hypotonia-seizures syndrome 3 (MCAHS3) is a rare genetic disorder, characterized by infantile-onset epilepsy, hypotonia, global developmental delay, dysmorphic features, and variable congenital anomalies involving the cardiac, skeletal, and genitourinary systems. It is caused by the homozygous or compound heterozygous mutation in the phosphatidylinositol glycan class T (PIGT) gene. Only fewer cases were reported in the literature till now. We described a PIGT mutation in an Indian girl with global developmental delay, infantile-onset seizures, hypotonia, and facial dysmorphism. This case will help to expand the clinical spectrum of PIGT mutation.

## Introduction

Multiple congenital anomalies-hypotonia-seizures syndrome type 3 (MCAHS3) is an autosomal recessive disorder characterized by infantile-onset epilepsy, hypotonia, global developmental delay, dysmorphic features, and variable congenital anomalies involving the cardiac, skeletal, and genitourinary systems [1]. It is caused by homozygous or compound heterozygous mutation in the phosphatidylinositol glycan class T (PIGT) gene on chromosome 20q13 [1]. The PIGT gene (OMIM: 610272) encodes the glycosylphosphatidylinositol (GPI) transamidase component of the PIGT enzyme, which catalyzes the attachment of proteins to GPI anchors and attaching the proteins to the cell membrane [2,3].

Till now, 14 patients with MCAHS3 disorder were reported in the literature. Here, we describe a homozygous PGIT mutation in an Indian girl with MCAHS3 disorder, which is helpful to expand the clinical spectrum of PIGT mutation disorders. 

## Case presentation

An 11-month-old Indian girl, the first child of a non-consanguineous couple, presented to the pediatric neurology clinic with complaints of seizures and global developmental delay. Pregnancy was uneventful. The child was born by cesarean section at term with a birth weight of 3 kg. At birth, the child was admitted to the neonatal intensive care unit with high total serum bilirubin (24.5 mg/dL) and treated with exchange transfusion and phototherapy. At that time, no other systemic complications were noticed. The child had a global developmental delay. At present, partial neck holding, no hand opening, no mother regard, and only making cooing sounds. The child had seizures three days before the visit to the clinic with an upper respiratory infection. In the last three days, the child had multiple episodes of generalized clonic seizures that last for the one-to-three minute duration.

**Figure 1:**
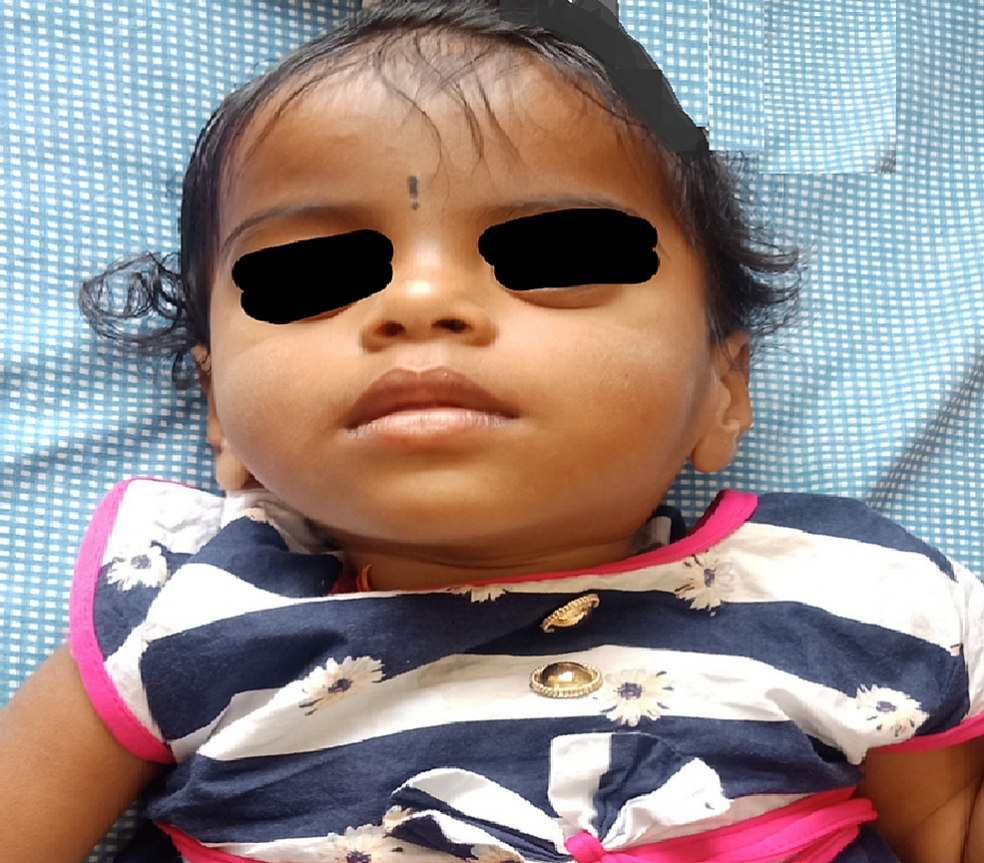
Dysmorphic face of the child

On examination, head circumference was 44 cm (25th centile), weight was 7 kg (<3rd centile) and height was 72 cm (25th centile). Physical examination showed dysmorphic features include frontal bossing, high forehead, depressed nasal bridge, low set ears, high arched palate, and long philtrum (Figure 1). Neurological examination revealed irritability, no visual fixation, nystagmus, central hypotonia, normal deep tendon reflexes, and choreoathetoid movements. The other systemic examination was normal. No skeletal anomalies were noticed.

**Figure 2:**
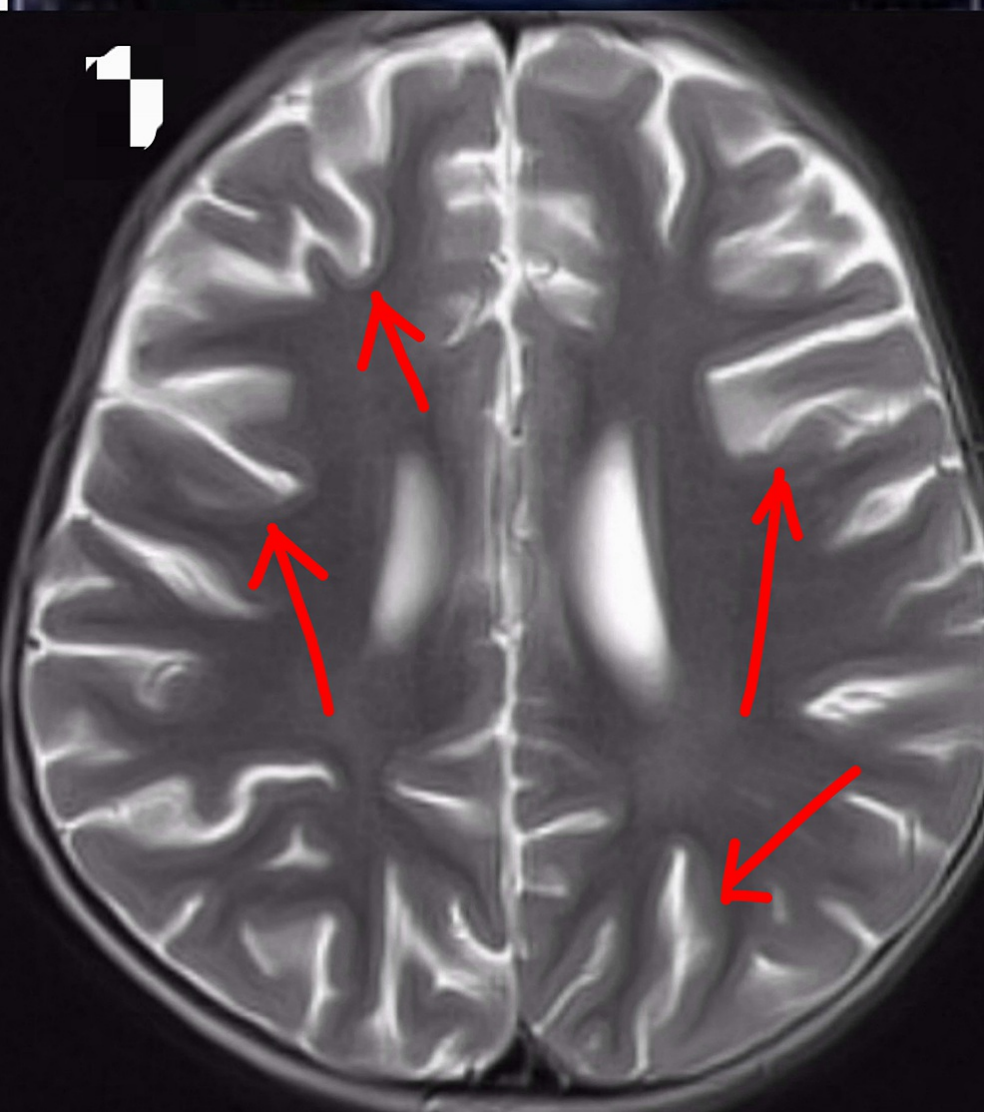
Magnetic resonance image of the brain T2 axial sequence showing features of cerebral atrophy.

Extensive investigations including complete blood picture, blood sugar, renal function tests, thyroid profile, lactate, creatine phosphokinase, alkaline phosphatase (ALP), vitamin B12, and vitamin D3 were normal. Both blood and urine amino acids and organic acids were unremarkable. Fundoscopy was normal. Cerebrospinal fluid analysis showed normal levels of glucose, proteins, lactate, and pyruvate. Abdominal ultrasonography and cardiac imaging were normal. Electroencephalography (EEG) revealed high amplitude diffuse slow waves background activity without any epileptiform discharges. MRI of the brain showed generalized atrophy without any focal lesions (Figure 2).

DNA sequencing with a next-generation sequencing platform revealed a homozygous missense variation in exon 10 of the PIGT gene (chr20: g.45424323C>T) that results in the amino acid substitution of tryptophan for arginine at codon 448 (p. Arg448Trp) was detected. This variant is classified as likely pathogenic in the ClinVar database.

Seizures were controlled with levetiracetam (20 mg/kg/day) and clobazam (0.7 mg/kg/day). Physiotherapy was initiated. Again child had seizures at 15 months of life with fever and controlled with an increased dose of levetiracetam (30 mg/kg/day). On follow-up, there were no further seizures. Now child's age was three years and completely bedridden, had feeding difficulties, and failure to thrive without recurrence of seizures.

## Discussion

Extracellular proteins are attached to the plasma membrane through a GPI anchor in humans. The transfer of GPI anchor to proteins carrying a C terminal GPI-attachment signal is a post-translational modification catalyzed by the GPI transamidase complex. This complex contains several crucial subunits, including the protein encoded by PIGT [2,3]. Mutations in the PIGT gene cause multiple congenital anomalies with neurological disorders known as MCAHS3 (OMIM # 615398). This MCAHS 3 is typically characterized by infantile-onset epilepsy, global developmental delay, hypotonia, craniofacial dysmorphic features, and congenital anomalies involving skeletal, ophthalmological, cardiac, and genitourinary systems [1].

Including the present report, 15 patients were reported with MCAHS 3 caused by PIGT mutations [1,4-9]. Among the reported six were male. Consanguinity was presented in some patients, but our patient was born to a non-consanguineous couple. Global developmental delay, infantile-onset seizures, cortical visual impairment, hypotonia, and facial dysmorphism were present in all the patients.

All the patients had infantile-onset seizures with various semiology including generalized tonic, clonic, focal and myoclonic seizures. These seizures are drug-resistant and needed multiple antiepileptic drugs to control. In the present report, the child had seizures at the age of 11months with fever but controlled with two antiepileptic drugs, which is a different feature compared to previous reports. These types of febrile-sensitive seizures were also reported by other studies [4-8]. Kohashi et al. reported an infant with recurrent epileptic apnea with resistant seizures [9].

In the present report, the child did not show skeletal, cardiac, and genitourinary abnormalities. Yang et al. and Skauli et al. also reported similar negative findings in those reports [5,8]. Almost all the studies reported nystagmus except Yang et al. [8].

Some studies showed low serum ALP, which supports the skeletal abnormalities and refractory seizures due to hypophosphatemia [1,6,7]. But along with our study, the other two studies reported normal ALP levels [4,5,8]. These findings indicate that low serum ALP cannot entirely explain the skeletal anomalies. MRI brain of the patient showed significant cerebral atrophy. Cerebral atrophy was reported in all the patients, which may be a key feature of this syndrome. Most of the children have cerebellar hypoplasia, which was not detected in this patient.

In this case, a homozygous mutation (g.45424323C>T, p. Arg448Trp) in the PIGT gene was detected. The same variant was reported by Lam et al and Nakashima et al, but those patients have compound heterozygous mutations along with this p.Arg448Trp [4,7]. The patients with the homozygous PIGT p.Thr183Pro were most severely affected [1]. But genotype-phenotype correlation needs further exploration.

In the present case, the child had severe neonatal jaundice and well-controlled seizures, which are not noticed in previous studies. This will help to further the expansion of the spectrum of PIGT mutation disorders.

## Conclusions

We report a homozygous PIGT (p.Arg448Trp) mutation in an Indian girl with global developmental delay, infantile-onset epilepsy, hypotonia, and facial dysmorphic features typical for MCAHS3. This patient did not show any skeletal, cardiac, and genitourinary abnormalities and had well-controlled febrile sensitive seizures. This report will help to expand the phenotypic spectrum of PIGT mutations.
